# CONTRABASS: exploiting flux constraints in genome-scale models for the detection of vulnerabilities

**DOI:** 10.1093/bioinformatics/btad053

**Published:** 2023-01-24

**Authors:** Alexandru Oarga, Bridget P Bannerman, Jorge Júlvez

**Affiliations:** Department of Computer Science and Systems Engineering, University of Zaragoza, Zaragoza 50018, Spain; Lucy Cavendish College, Biological Sciences, University of Cambridge, Cambridge CB3 0BU, UK; Science Resources Foundation, Health Unit, London EC1V 2NX, UK; Department of Computer Science and Systems Engineering, University of Zaragoza, Zaragoza 50018, Spain

## Abstract

**Motivation:**

Despite the fact that antimicrobial resistance is an increasing health concern, the pace of production of new drugs is slow due to the high cost and uncertain success of the process. The development of high-throughput technologies has allowed the integration of biological data into detailed genome-scale models of multiple organisms. Such models can be exploited by means of computational methods to identify system vulnerabilities such as chokepoint reactions and essential reactions. These vulnerabilities are appealing drug targets that can lead to novel drug developments. However, the current approach to compute these vulnerabilities is only based on topological data and ignores the dynamic information of the model. This can lead to misidentified drug targets.

**Results:**

This work computes flux constraints that are consistent with a certain growth rate of the modelled organism, and integrates the computed flux constraints into the model to improve the detection of vulnerabilities. By exploiting these flux constraints, we are able to obtain a directionality of the reactions of metabolism consistent with a given growth rate of the model, and consequently, a more realistic detection of vulnerabilities can be performed. Several sets of reactions that are system vulnerabilities are defined and the relationships among them are studied. The approach for the detection of these vulnerabilities has been implemented in the Python tool CONTRABASS. Such tool, for which an online web server has also been implemented, computes flux constraints and generates a report with the detected vulnerabilities.

**Availability and implementation:**

CONTRABASS is available as an open source Python package at https://github.com/openCONTRABASS/CONTRABASS under GPL-3.0 License. An online web server is available at http://contrabass.unizar.es.

**Supplementary information:**

A glossary of terms are available at *Bioinformatics* online.

## 1 Introduction

Antimicrobial resistance (AMR) occurs when bacteria, viruses, fungi and parasites evolve to become unresponsive to conventional antibiotics, thus threatening our ability to treat common infections and increasing the risk of spread of severe illnesses. The emergence of multidrug-resistant bacteria (MDR) is particularly alarming, as they can cause infections untreatable with existing antibiotics. According to the World Health Organization, AMR is one of the primary global public health threats of humanity due to its high increasing rate (Leung *et al.*, 2011).

Despite the increasing emergency of AMR, the pipeline of drug development involves a huge cost of time and budget and, in addition, most drug candidates fail the clinical stages. Therefore, novel approaches for the development of drugs that tackle the rising problem of AMR should be established urgently (Jubeh *et al.*, 2020).

The emergence of high-throughput technologies has led to the construction of large datasets with structural and transcriptomic data of different pathogens. The integration of multidimensional data has the potential to offer a more rapid and cost-effective strategy compared to traditional screening methods in the process of drug discovery. In this context, the integration of metabolic functions obtained from genome annotations enabled the reconstruction of metabolic networks of multiple microorganisms. These reconstructions allow the modelling of metabolism, which can provide non-intuitive insights into biological systems that *in vivo* assays alone cannot provide (Ramos *et al.*, 2018; Richelle *et al.*, 2020).

Metabolism is the set of basic life processes that take place in the cell, and it is the means by which cells can maintain life and grow from their environment. Metabolism can be represented as a metabolic network, which includes all the metabolic reactions that can occur in a cell. A possible strategy for drug development is to find vulnerabilities in the metabolism that can stop the growth and replication of the bacterium, and thus its damaging effects.

As of 2019, genome-scale models (GEMs) of metabolism have been reconstructed for more than 6000 organisms including bacteria, archaea and eukaryotes by either automatic or manual reconstruction (Gu *et al.*, 2019). GEMs have proven useful in a wide range of applications, such as expanding knowledge on microorganisms (Henry *et al.*, 2006; Navid and Almaas, 2012) and microbial communities (Kumar *et al.*, 2019; Zomorrodi and Maranas, 2012), microbial engineering (McAnulty *et al.*, 2012; Roberts *et al.*, 2010) and drug discovery (Ramos *et al.*, 2018). Furthermore, these models have proven to be interesting in areas such as oncology, by studying drug targets in cancer metabolism (Folger *et al.*, 2011), and viral diseases (Bannerman *et al.*, 2021). Drug targeting in pathogens is usually performed by considering essential genes, reactions or metabolites, whose inhibition can effectively kill a pathogen (Gu *et al.*, 2019) or through metabolic network topology analysis (Ramos *et al.*, 2018).

Despite the number of GEMs developed recently, most of these models lack genetic information, which could hamper the model analysis based on gene essentiality and calls for the design of computational methods that exploit as much as possible the available biological information. Here, we focus on the computation of critical reactions in metabolic networks such as essential reactions, dead reactions, and chokepoints (Chukualim *et al.*, 2008; Yeh *et al.*, 2004).

A reaction is essential if it is required by the organism to grow. The deletion of one of these reactions has the potential to cause a malfunction in the metabolism and stop the growth of the organism. As this eventually leads to the death of the organism, essential reactions are widely accepted as drug target candidates (Oyelade *et al.*, 2018).

Dead reactions are those reactions that cannot carry any flux. These reactions will receive special attention since they may reflect an incompleteness of the model or non-preferred pathways of the organism. As it will be shown, these reactions directly affect the number of critical reactions computed for a model.

A chokepoint is a reaction that is the only consumer or the only producer of a given metabolite. Thus, the inhibition of a chokepoint may lead to the unlimited accumulation of potentially toxic metabolites or the lack of production of an essential compound. Chokepoints are, therefore, appealing drug targets in microbial metabolism.

The current methods for the computation of chokepoints only make use of the topological properties of the metabolic network. Although the directionality of the network accounts for the sets of reactants and products of each reaction, it disregards the fact that such sets depend on the fluxes of the reactions.

Reaction fluxes define the direction in which they take place. To establish a particular direction, all reactions are given flux bounds that delimit the range of flux values that a reaction can take. Reactions that are given positive upper flux bounds and negative lower flux bounds are said to be reversible. These flux bounds are used to model reactions that can take place in both directions or simply to model reactions whose direction is unknown (Thiele and Palsson, 2010). For instance, let us say that a chemical reaction r:A↔B is reversible. This means that *r* can happen in a forward direction (r:A→B) when a positive flux is assigned, or a backward direction when a negative flux is assigned (r:A←B). Notice that this means that metabolites in reversible reactions can act both as reactants and as products. In the reaction r:A→B, metabolite A  is the reactant and metabolite *B* is the product, however, if the flux of the reaction is negative, then *A* becomes the product and *B* the reactant.

In most GEMs, unknown flux bounds are given default flux bounds, e.g. $b(r)=-1000\;\text{mmol}\;{\text{gDW}}^{-1}h^{-1}$ and $\text{ub}(r)=1000\;\text{mmol}\;{\text{gDW}}^{-1}h^{-1}$, that is, reactions are defined as reversible by default. However, not all these default flux bounds are compatible with a positive growth rate. This work exploits the flux constraints of the model to compute sets of reactants and products that are consistent with a given growth rate, and in turn, to compute growth-dependent critical reactions. Such approach improves the detection of vulnerabilities and is applied to compute chokepoints, essential reactions and dead reactions. The dependence of these sets with respect to growth is also inferred theoretically. To facilitate the computation of vulnerabilities in GEMs by integrating dynamic constraints, a software tool (*CONTRABASS*) has been developed.

The remainder of the article is organized as follows: Sections 2.1 and 2.2 recall preliminary concepts and definitions such as constraint-based models and essential reactions. Structural definitions of constraint-based models and flux-dependent definitions are recalled in Sections 2.3 and 2.4, respectively. In Section 2.5, growth-dependent definitions are introduced. Section 2.6 is devoted to dead reactions, blocked reactions and their relationship. Section 2.7 shows how the integration of dynamic constraints affects the computation of vulnerabilities in *Plasmodium falciparum* model. Finally, Section 3 reviews the implemented tool *CONTRABASS* and Section 4 concludes the article.

## 2 Systems and methods

### 2.1 Constraint-based models

This subsection recalls definitions (Oarga *et al.*, 2020) and methods that will be used in the next sections.

Definition 2.1. A *constraint-based model* (Orth *et al.*, 2011; Varma and Palsson, 1994) is a tuple {R, M, S, L, U} where R is a set of reactions, M is a set of metabolites, $S\in R^{\left|M|\times |R\right|}$ is the stoichiometric matrix, and $L,U\in R^{\left|R\right|}$ are lower and upper flux bounds of the reactions.

Without loss of generality, it is assumed that L[r]≤U[r]  ∀r∈R.

All reactions are associated with a set of reactant metabolites and a set of product metabolites (one of these two sets can be empty). For example, the reaction r:A→2B has a reactant metabolite *A*, and a product metabolite *B* with stoichiometric weight 2, i.e. two molecules of type *B* are produced per each molecule of type *A* that is consumed by *r*. The stoichiometric matrix S accounts for all the stoichiometric weights of the reactions, i.e. S[m,r] is the stoichiometric weight of metabolite m∈M for reaction r∈R. Thus,

- if S[m,r]<0 then *m* is a reactant and is consumed when *r* occurs.
- if S[m,r]>0 then *m* is a product and is produced when *r* occurs.
- if S[m,r]=0 then *m* is neither consumed nor produced when *r* occurs.

Constraint-based models can be expressed as bipartite graphs with two types of nodes representing reactions and metabolites. Hence, constraint-based models can be represented graphically as Petri nets (Heiner *et al.*, 2008; Murata, 1989), where places, drawn as circles, model metabolites, and transitions, drawn as squares, model reactions. The presence of an arc from a place (transition) to a transition (place) means that the place is a reactant (product) of the reaction modelled by the transition. The weights of the arcs of the Petri net account for the stoichiometry of the constraint-based model. In other words, the stoichiometric matrix of a constraint-based model and the incidence matrix of its corresponding Petri net coincide.

**Fig. 1.**
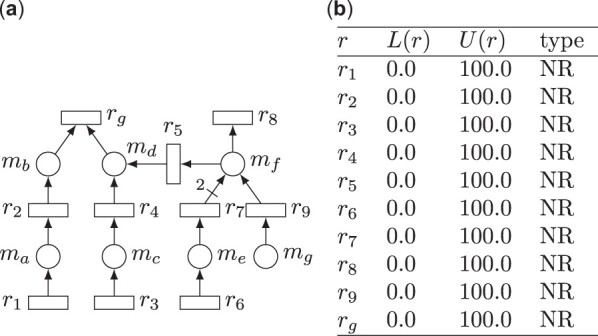
(**a**) Example Petri net modelling a constraint-based model. (**b**) Flux bounds of the model

Example 2.1. The Petri net in Figure 1 represents a simple constraint-based model that consists of 10 reactions and 7 metabolites. As an example, transition *r*_7_ models the reaction $r_{7}:m_{e}\to 2m_{f}$.

Flux balance analysis (FBA) (Orth *et al.*, 2010) is a mathematical procedure for the estimation of steady-state fluxes in constraint-based models. Among other possibilities, FBA can be used to predict the growth rate of an organism or the rate of production of a given metabolite.

Let $v\in R^{\left|R\right|}$ be the vector of fluxes of reactions and v[r] denote the flux of reaction *r*. The system is assumed to be mass balanced at steady state, this is, the rate of production and rate of consumption of metabolites is constant. This constraint is given by the expression S·v=0, where *S* is the stoichiometric matrix. The steady-state mass balance fluxes of reactions, *v*, must also satisfy the lower and upper bounds *L* and *U*. Thus, the linear programming problem (LPP) for FBA is:

(1) $$\begin{array}{cc} \max\; & z\cdot v\\ \text{st}.\;\; & S\cdot v=0\\ & L\leq v\leq U \end{array}$$

where $z\in R^{\left|R\right|}$ expresses the objective function.

Let *r*_g_ be the reaction that models growth (or biomass production) in a constraint-based model. Without loss of generality, it will be assumed that $L[r_{g}]\geq 0$. An optimistic estimate for the growth rate of the modelled microorganism can be obtained by:

(2) $$\begin{array}{cc} \max\; & v[r_{g}]\\ \text{st}.\;\; & S\cdot v=0\\ & L\leq v\leq U \end{array}$$

The solution of the above LPP (Equation (2)) will be denoted by *μ*_max_.

### 2.2 Essential reactions

A reaction is said to be essential if it is required by the organism to grow. In other words, the deletion of an essential reaction implies null growth. Consequently, these reactions have the potential to cause the death of the modelled organism.

Definition 2.2. (Oyelade *et al.*, 2018) A reaction r∈R is an *essential reaction* if the solution of the following LPP:

(3) $$\begin{array}{cc} \max\; & v[r_{g}]\\ \text{st}.\;\; & S\cdot v=0\\ & L\leq v\leq U\\ & v[r]=0 \end{array}$$

is equal to 0 or the LPP is infeasible.

The set of essential reactions, which is denoted ER, can be computed straightforwardly by solving Equation (3) for each r∈R.

Example 2.2. Let us say that in the Petri net of Figure 1 reaction *r*_g_ models growth. In this model, reactions *r*_1_ and *r*_2_ are essential reactions. This is because, if the flux of any of these reactions is set to 0, then it is not possible to produce metabolite *m*_b_, which is necessary for the growth reaction *r*_g_.

### 2.3 Structural definitions

This section recalls how structural definitions of chokepoints and dead-end metabolites can be defined by making use of Petri net notation. Let *X* denotes a vertex of the net, i.e. a reaction or a metabolite. Then, ${}^{•}X$($X^{•}$) denotes the set of input (output) vertices of *X*. For instance, for a given reaction $r\in R,\;{}^{•}r$ denotes its set of reactants and $r^{•}$ its set of products; for a given metabolite $m\in M,\;{}^{•}m$ denotes its set of producing reactions and $m^{•}$ its set of consuming reactions.

A chokepoint is a reaction that is the only producer or the only consumer of a metabolite. More formally:

Definition 2.3. (Chukualim *et al.*, 2008; Yeh *et al.*, 2004) A reaction r∈R is a chokepoint if there exists m∈M such that $m^{•}=\{r\}$ or ${}^{•}m=\{r\}$.

The set of chokepoint reactions will be denoted as CP. Notice that the inhibition of a chokepoint reaction can lead either to the infinite accumulation of a substrate (which will not be used as expected or might be toxic) or to the depletion of a metabolite (which might be essential for the cell) (Chukualim *et al.*, 2008; Yeh *et al.*, 2004). The identification of chokepoints is, therefore, relevant to the identification of potential drug targets.

A dead-end metabolite is a metabolite without input or output reactions:

Definition 2.4. (Mackie *et al.*, 2013) A metabolite m∈M is a dead-end metabolite if $m^{•}=\{\}$ or ${}^{•}m=\{\}$.

Dead-end metabolites can indicate a potential shortcoming or incompleteness of the model since their concentration can only increase or decrease.

### 2.4 Flux-dependent definitions

The previous definitions only take into account the structure of the network and disregard the flux bounds of the reactions. In order to capture the fact that reactions can proceed forwards or backwards, e.g. reversible reactions, new sets of reactants, products, consumers and producers that take into account flux bounds were defined (Oarga *et al.*, 2020):

- Flux-dependent set of reactants of *r*:
  $${}^{⋆}r=\{m\in M|(S(m,r)<0\wedge U[r]>0)\vee (S(m,r)>0\wedge L[r]<0)\}$$
- Flux-dependent set of products of *r*:
  $$r^{⋆}=\{m\in M|(S(m,r)>0\wedge U[r]>0)\vee (S(m,r)<0\wedge L[r]<0)\}$$
- Flux-dependent set of producers of *m*:
  $${}^{⋆}m=\{r\in R|m\in r^{⋆}\}$$
- Flux-dependent set of consumers of *m*:
  $$m^{⋆}=\{r\in R|m\in {}^{⋆}r\}$$

Flux-dependent chokepoints and dead-end metabolites can be defined accordingly (Oarga *et al.*, 2020):

Definition 2.5. A reaction r∈R is a flux-dependent chokepoint if there exists m∈M such that $m^{⋆}=\{r\}$ or ${}^{⋆}m=\{r\}$. The set of flux-dependent chokepoints is denoted ${\text{CP}}_{⋆}$.

Definition 2.6. A metabolite m∈M is a flux-dependent dead-end metabolite if $m^{⋆}=\{\}$ or ${}^{⋆}m=\{\}$. The set of flux-dependent dead-end metabolites is denoted ${\text{DEM}}_{⋆}$.

Example 2.3. In the Petri net in Figure 1, *r*_1_ is a producer of *m*_a_, i.e. $r_{1}\in {}^{•}m_{a}$; *r*_2_ is a flux-dependent chokepoint because it is the only consumer of *m*_a_, i.e. $m_{a}{}^{•}=\{r_{2}\}$ and $r_{2}\in {\text{CP}}_{⋆}$; and *m*_g_ is a flux-dependent dead-end metabolite, i.e. $m_{g}\in {\text{DEM}}_{⋆}$.

In addition to chokepoints, flux bounds can be used to classify reactions as *dead*, *reversible* or *non-reversible*:

Definition 2.7. A reaction r∈R is dead if L[r]=U[r]=0.

Definition 2.8. A reaction r∈R is reversible if L[r]<0<U[r].

Definition 2.9. A reaction r∈R is non-reversible if *r* is not dead and *r* is not reversible.

From the above definitions, it can be deduced that *r* is non-reversible if (0≤L[r]∧0<U[r]) ∨(L[r]<0∧U[r]≤0).

Notice that dead reactions will never have a non-zero flux. Since this might indicate a deficiency of the model or a blocked pathway of the organism, special attention will be paid to dead reactions.

The sets of dead, reversible and non-reversible reactions are denoted DR, RR and NR, respectively. By definition, DR, RR and NR represent a partition of the set of reactions R, i.e. DR∪RR∪NR=R, DR∩RR=∅, RR∩NR=∅ and DR∩NR=∅.

For the Petri net representation of a constraint-based model, dead reactions, reversible reactions and non-reversible reactions will be represented as rectangles with a cross inside, as double rectangles, and as rectangles, respectively.

**Fig. 2.**
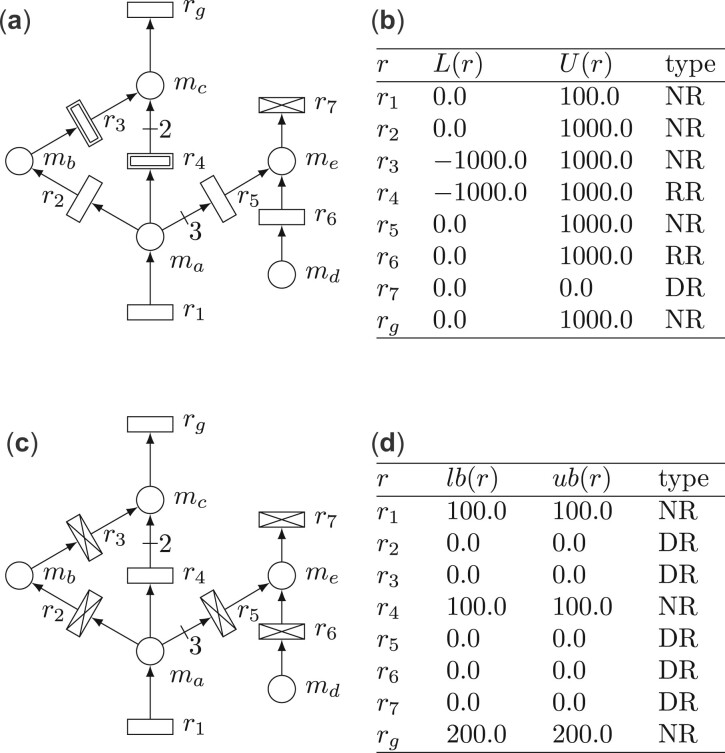
(**a**) Petri net modelling a constraint-based model. (**b**) Initial flux bounds. (**c**) Petri net modelling a constraint-based model obtained with γ = 1 and *r*_g_ as objective. (**d**) Constrained flux bounds obtained by FVA with γ = 1

Example 2.4. The Petri net in Figure 2a represents a constraint-based model with five metabolites and eight reactions. In this model, reactions *r*_3_, *r*_4_ are reversible reactions (i.e. $\text{RR}=\{r_{3},r_{4}\}$), reaction *r*_7_ is a dead reaction (i.e. $\text{DR}=\{r_{7}\}$) and reactions $r_{1},r_{2},r_{5},r_{6},r_{g}$ are non-reversible reactions (i.e. $\text{NR}=\{r_{1},r_{2},r_{5},r_{6},r_{g}\}$).

### 2.5 Growth-dependent definitions

In contrast to the previous flux-dependent definitions of reactions, this section introduces growth-dependent definitions, i.e. the sets of reactions that depend on the growth constraints that are imposed on the model.

#### 2.5.1 Essential reactions

Similar to essential reactions, which are those reactions that are necessary to produce non-null growth on the model, growth-dependent essential reactions are those reactions that are necessary to produce a certain growth on the model. This certain growth will be expressed as ${\gamma \cdot \mu }_{\text{max}}$ where γ∈[0,1] and *μ*_max_ is the solution of Equation (2).

A reaction is said to be a growth-dependent essential reaction for a given growth ${\gamma \cdot \mu }_{\text{max}}$ if its deletion implies that the maximum possible growth is below ${\gamma \cdot \mu }_{\text{max}}$. More formally,

Definition 2.10. Let *μ*_max_ be the solution of the LPP in Equation (2). Given γ∈[0,1], a reaction r∈R is a *growth-dependent essential reaction* if the solution of LPP (Equation (3)) is lower than ${\gamma \cdot \mu }_{\text{max}}$ or the LPP is infeasible.

The set of growth-dependent essential reactions for a given growth specified by γ∈[0,1] will be denoted ${\text{ER}}_{\gamma }$. This set can be computed straightforwardly by solving LPP (Equation (3)) for each reaction.

Special attention is given to the set of reactions ER_1_, as it will consist of those reactions that are necessary to produce the optimal growth of the model. This set will be named *essential reactions for optimal growth* (EROG).

Example 2.5. In the Petri net of Figure 1 where *r*_g_ models growth, reactions $r_{1},r_{2},r_{3},r_{4}$ are EROG (i.e. $r_{1},r_{2},r_{3},r_{4}\in \text{EROG}$). Reactions *r*_1_, *r*_2_ are essential reactions, and thus, they are also EROG. If one, or both reactions, *r*_3_, *r*_4_ are forced to have flux equal to 0, metabolite *m*, which is essential for growth, can only be produced through a non-optimal path. Then, the model will not be able to achieve optimal growth. Therefore, these reactions are EROG.

#### 2.5.2 Dead, reversible and chokepoint reactions

Flux variability analysis (FVA) (Mahadevan and Schilling, 2003) is a mathematical procedure to compute the minimum and maximum fluxes of reactions that are compatible with some state. Let *μ*_max_ be the maximum growth calculated by FBA (Equation (2)). FVA is computed by solving two independent LPPs per reaction r∈R. One programming problem maximizes the flux of *r*, v[r], and the other minimizes v[r]. The constraints of both problems are the same: the steady state condition S·v=0, the flux bounds L[r]≤v[r]≤U[r], and the maintenance of *μ*_max_ to a certain degree γ∈[0,1]. The two programming problems for a given reaction r∈R can be expressed as:

(4) $$\begin{array}{cc} \max/\min\; & v[r]\\ \text{st}.\;\; & S\cdot v=0\\ & L\leq v\leq U\\ & {\gamma \cdot \mu }_{\text{max}}\leq v[r_{g}] \end{array},$$

where *r*_g_ is the reaction that models growth.

The computation of the flux bounds by means of FVA (Equation (4)), can be carried out in an optimal state, i.e. γ = 1, or in a suboptimal state, i.e. 0≤γ<1. In the optimal state, all fluxes must be optimally directed towards growth, whereas in suboptimal states, fluxes are allowed to deviate towards other functionalities.

Let $lb_{\gamma },ub_{\gamma }\in R^{\left|R\right|}$ be the result of computing FVA (Equation (4)) on a constraint-based model {R, M, S, L, U} for a given γ, i.e. $lb_{\gamma }[r]$ and $ub_{\gamma }[r]$ are the minimum and maximum fluxes given by FVA for reaction *r*. If the flux bounds *L*, *U* of the constrained-based model are replaced by $lb_{\gamma },ub_{\gamma }$, a new constraint-based model, $\left\{R,\;M,\;S,\;lb_{\gamma },\;ub_{\gamma }\right\}$, with more restrictive (and realistic) flux bounds is obtained.

Given γ∈[0,1], the sets of growth-dependent products, reactants, consumers, and producers of the model {R, M, S, L, U}, which are denoted $r^{\gamma },{}^{\gamma }r,m^{\gamma },{}^{\gamma }m$, respectively, are defined as the flux-dependent products, reactants, consumers and products of $\left\{R,\;M,\;S,\;lb_{\gamma },\;ub_{\gamma }\right\}$ as discussed in Section 2.4.

Similarly, given {R, M, S, L, U} and γ∈[0,1], the sets of growth-dependent dead, reversible and non-reversible reactions, which are denoted ${\text{DR}}_{\gamma },\;{\text{RR}}_{\gamma }$ and ${\text{NR}}_{\gamma }$, the sets of growth-dependent chokepoint reactions and dead-end metabolites, which are denoted as ${\text{CP}}_{\gamma }$ and ${\text{DEM}}_{\gamma }$, are also defined as the corresponding flux-dependent elements of $\left\{R,\;M,\;S,\;lb_{\gamma },\;ub_{\gamma }\right\}$.

Example 2.6. Let us assume that *r*_g_ in Figure 2a represents growth, i.e. the component of *z* in Equation (1) that corresponds to *r*_g_ is equal to 1 and the rest of components of *z* are 0. In order to obtain growth-dependent sets, the flux bounds computed by FVA with *γ* = 1 are assigned to the reactions and the net in Figure 2c is obtained (notice that other values of *γ* can be considered). In this new net, *r*_2_, *r*_3_, *r*_5_, *r*_6_ and *r*_7_ are dead reactions, i.e. $r_{2},r_{3},r_{5},r_{6},r_{7}\in {\text{DR}}_{1}$, and *r*_4_, which was a reversible reaction in Figure 2a, becomes a non-reversible reaction, i.e. $r_{4}\in {\text{NR}}_{1}$.

In the model of Figure 2a, reaction *r*_3_ is the only flux-dependent consumer of metabolite *m*_b_ and, thus, is a flux-dependent chokepoint reaction, i.e. $r_{3}\in {\text{CP}}_{⋆}$. However, in the growth-dependent model of Figure 2c, *r*_3_ becomes a dead reaction, i.e. $r_{3}\in {\text{DR}}_{1}$, and hence, it is no longer considered a chokepoint reaction, i.e. $r_{3}\notin {\text{CP}}_{1}$.

On the other hand, reaction *r*_4_ is not the only producer or consumer of any metabolite in the model of Figure 2a, and thus, it is not a flux-dependent chokepoint reaction, i.e. $r_{4}\notin {\text{CP}}_{⋆}$. However, in the growth-dependent model of Figure 2c, reactions *r*_2_, *r*_3_ and *r*_5_ become dead reactions and reaction *r*_4_ becomes the only consumer of *m*_a_ and the only producer of *m*_c_, consequently, *r*_4_ becomes a growth-dependent chokepoint reaction $r_{4}\in {\text{CP}}_{1}$.

Given their importance in model tuning and drug discovery, the following sections focus on dead reactions, essential reactions and chokepoint reactions.

### 2.6 Dead reactions

This subsection explores the relationship between dead reactions and blocked reactions, and shows that the set of growth-dependent dead reactions is the same for any suboptimal state, i.e. for any growth strictly lower than the maximum growth *μ*_max_. Such a set coincides with the set of blocked reactions. Recall that a reaction r∈R is said to be blocked if its flux is 0 at any possible steady state. More formally:

Definition 2.11. (Vlassis *et al.*, 2014) A reaction r∈R is a blocked reaction if for every $v\in R^{\left|R\right|}$ such that S·v=0, it holds v[r]=0.

Example 2.7. In the Petri net in Figure 2a, reaction *r*_6_ is a blocked reaction. This is because *r*_6_ consumes *m*_d_, which is a DEM. If the flux of *r*_6_ were positive (negative), the amount of *m*_d_ would decrease(increase) indefinitely, which contradicts the steady-state constraint, therefore the only possible steady-state flux for *r*_6_ is $v[r_{6}]=0$.

In Vlassis *et al.* (2014) blocked reactions are obtained by solving the following LPPs that compute the maximum and minimum feasible fluxes of the reactions subject to the steady state constraint S·v=0:

(5) $$\begin{array}{cc} \max/\min\;\; & v[r]\\ \text{st}.\;\; & S\cdot v=0\\ & L\leq v\leq U \end{array}$$

A reaction with null maximum and minimum feasible flux is a blocked reaction. Note that this procedure is equivalent to computing FVA, see Equation (4), with γ = 0. Thus, the set of blocked reactions is equal to DR_0_.

Interestingly, the set of growth-dependent dead reactions, ${\text{DR}}_{\gamma }$ is the same for any γ such that 0≤γ<1. In other words, the set of dead reactions in suboptimal states, regardless of the growth rate imposed on the model, is equivalent to the set of blocked reactions. This fact will be proved through several steps. Let us first prove that the range of feasible fluxes of a reaction *r*, i.e. the interval $\left[lb_{\gamma }[r],ub_{\gamma }[r]\right]$, cannot increase as γ increases.

Lemma 2.1. $[lb_{\gamma_{2}}[r],ub_{\gamma_{2}}[r]]\subseteq [lb_{\gamma_{1}}[r],ub_{\gamma_{1}}[r]]\;\forall \;r\in R$∧$\forall \;\gamma_{1}$, γ_2_*such that* $0\leq \gamma_{1}<\gamma_{2}\leq 1$.

Proof.

Given γ∈[0,1], the range of feasible fluxes of r∈R, $\left[lb_{\gamma }[r],ub_{\gamma }[r]\right]$, is given by the solutions of FVA (Equation (4)). Notice that the constraints of such LPP define a convex set of possible solutions which can only shrink as γ increases, i.e. as the constraint ${\gamma \cdot \mu }_{\text{max}}\leq z\cdot v$ becomes more restrictive. Thus, if $\gamma_{1}<\gamma_{2}$ then $lb_{\gamma_{1}}[r]\leq lb_{\gamma_{2}}[r]$ and $ub_{\gamma_{1}}[r]\geq ub_{\gamma_{2}}[r]$. □

Then, the set of growth-dependent dead reactions cannot decrease with γ:

Lemma 2.2. ${\text{DR}}_{\gamma_{1}}\subseteq {\text{DR}}_{\gamma_{2}}\;\forall \;\gamma_{1}$, γ_2_*such that* $0\leq \gamma_{1}<\gamma_{2}\leq 1$.

Proof. Let $r\in {\text{DR}}_{\gamma_{1}}$, i.e. $lb_{\gamma_{1}}[r]=ub_{\gamma_{1}}[r]=0$, then by Lemma 2.1, it follows that $lb_{\gamma_{2}}[r]=ub_{\gamma_{2}}[r]=0$ and hence $r\in {\text{DR}}_{\gamma_{2}}$. □

Let us now show that the set of growth-dependent dead reactions cannot increase with γ in suboptimal states, i.e. with γ<1:

Lemma 2.3. ${\text{DR}}_{\gamma_{1}}⊇{\text{DR}}_{\gamma_{2}}\;\forall \;\gamma_{1}$, γ_2_*such that* $0\leq \gamma_{1}<\gamma_{2}<1$.

Proof. The convex set of possible solutions defined by the constraints in Equation (4) can only decrease as γ increases, see Lemma 2.1. Assume there exist $\gamma_{a},\gamma_{b}$ such that $0\leq \gamma_{a}<\gamma_{b}<1,\;lb_{\gamma_{a}}[r]<ub_{\gamma_{a}}[r]$ and $lb_{\gamma_{b}}[r]=ub_{\gamma_{b}}[r]=0$, i.e. *r* becomes dead when γ_a_ is increased to γ_b_. Then, given that the constraints in Equation (4) are linear, if γ_b_ is further increased by ϵ∈R such that ϵ>0 and $\gamma_{b}+\epsilon <1$, then $lb_{\gamma_{b}+\epsilon }[r]>ub_{\gamma_{b}+\epsilon }[r]$ should hold which is not possible because a lower bound cannot exceed an upper bound. □

From Lemmas 2.2 and 2.3, the following theorem can be derived straightforwardly:

Theorem 2.4. ${\text{DR}}_{\gamma_{1}}={\text{DR}}_{\gamma_{2}}\;\forall \gamma_{1},\gamma_{2}\in [0,1)$.

Thus, in particular, the set of blocked reactions coincides with the set of dead reactions in suboptimal states.

Corollary 2.5. ${\text{DR}}_{\gamma }={\text{DR}}_{0}\;\forall \gamma \in [0,1)$.

**Fig. 3.**
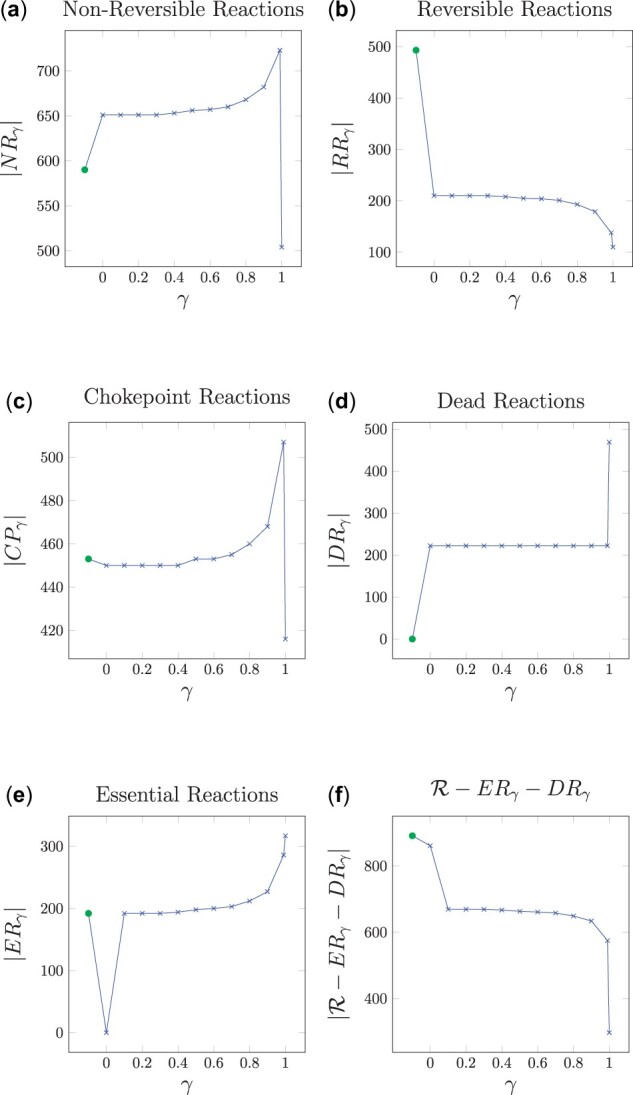
Sizes of the sets of reactions ${\text{NR}}_{\gamma },\;{\text{RR}}_{\gamma },\;{\text{CP}}_{\gamma },\;{\text{DR}}_{\gamma },\;{\text{ER}}_{\gamma }$ and $R-{\text{ER}}_{\gamma }-{\text{DR}}_{\gamma }$ of *P.falciparum* for γ∈[0,1]. The leftmost value of each plot corresponds to NR, RR, ${\text{CP}}_{⋆}$, DR, ER and R−ER−DR, respectively

Notice that DR_1_ can be strictly greater than ${\text{DR}}_{\gamma }$ with γ∈[0,1). As an example, the next subsection analyses a constrained-based model in which DR_1_ is strictly greater than ${\text{DR}}_{\gamma }$ with γ∈[0,1), see Figure 3d.

### 2.7 Case study: *Plasmodium falciparum*

This section presents the results obtained for the constraint-based model of *P.falciparum* (iAM-Pf480) (Abdel-Haleem *et al.*, 2018). The model includes a total of 1083 reactions, 909 metabolites, and 480 genes. The sizes of the sets of flux-dependent reactions are |RR|=493,|NR|=590,|DR|=0. The number of initial flux-dependent chokepoint reactions is |${\text{CP}}_{⋆}$| = 453. The sets of essential reactions and EROG were also computed, and the following sizes were obtained: |ER|=192 and |EROG|=317.

Figure 3 shows the sizes of the growth-dependent sets ${\text{NR}}_{\gamma },\;{\text{RR}}_{\gamma },\;{\text{CP}}_{\gamma },\;{\text{DR}}_{\gamma },\;{\text{ER}}_{\gamma },\;R-{\text{ER}}_{\gamma }-{\text{DR}}_{\gamma }$ in plots 3(a), 3(b), 3(c), 3(d), 3(e) and 3(f), respectively. To assess the impact of γ on these sets, different values of γ in the interval [0,1] have been used. In addition to the sizes of the sets obtained with γ, the leftmost value (depicted in green) of plots 3(a), 3(b), 3(c) and 3(d) refers to the size of the flux-dependent set prior to FVA, i.e. NR, RR,${\text{CP}}_{⋆}$ and DR. Similarly, the leftmost value (in green) of plots 3(e) and 3(f) includes the sizes of the set of essential reactions ER and the set R−ER−DR, respectively.

Notice that if γ = 0, the constraint ${\gamma \cdot \mu }_{\text{max}}\leq z\cdot v$ in Equation (4) does not impose a minimum growth on the model, and only the steady state condition S·v=0 must be satisfied. It can be seen that at γ = 0, the set of dead reactions exhibits an increase from the initial set of flux-dependent dead reactions: |DR|=0 and $|{\text{DR}}_{0}|=222$. As noted in Section 2.6, this set DR_0_ is equal to the set of blocked reactions, and keeps the same for every γ∈[0,1), see Figure 3d. The second increase in the set of dead reactions takes place at γ = 1 and it is due to the fact that in the optimal growth state, the flux must be necessarily distributed through optimal paths for biomass production and no flux can be diverted through other paths. Thus, non-optimal reactions for biomass production become dead reactions.

With respect to reversible reactions, the steady-state constraint S·v=0 reduces the size of this set from |RR|=493 to $|{\text{RR}}_{0}|=210$, see Figure 3b. Such a reduction is caused by the blocked reactions that belonged to RR and become dead reactions, and by the reversible reactions that become non-reversible with the steady-state constraint.

Similarly, blocked reactions that belonged to the set of flux-dependent non-reversible reactions NR become dead reactions with the steady-state constraint. However, due to the significant amount of reversible reactions that become non-reversible reactions, the size of the set increases from |NR|=590 to $|{\text{NR}}_{0}|=651$, see Figure 3a.

With regard to chokepoint reactions, see Figure 3c, the number of flux-dependent chokepoints is $|{\text{CP}}_{⋆}|=453$. At γ = 0, there is a decrease to $|{\text{CP}}_{0}|=450$, and then the set increases slowly until $|{\text{CP}}_{0.99}|=507$. As in the sets of non-reversible reactions and reversible reactions, the set of chokepoints decreases at γ = 1 as many reactions become dead reactions.

Notice that the set of chokepoints at γ = 1, CP_1_, is smaller than the set of flux-dependent chokepoints, ${\text{CP}}_{⋆}$. Moreover, CP_1_ is not contained in ${\text{CP}}_{⋆}$. This is due to the changes produced in the sets of non-reversible reactions and reversible reactions as γ increases. Recall that a reversible reaction is considered simultaneously both as a consumer and as a producer of a given metabolite. If this reaction becomes non-reversible, then it might not be a producer and consumer of a given metabolite. This fact can lead to the appearance of new growth-dependent chokepoints in CP_1_ that were not chokepoints in ${\text{CP}}_{⋆}$. On the other hand, dead reactions are neither consumers nor producers and, hence, they are not chokepoints. Thus, the increase of dead reactions in DR_1_ can imply changes in CP_1_ with respect to ${\text{CP}}_{⋆}$.

Figure 3e reports the size of the set of essential reactions ${\text{ER}}_{\gamma }$ with respect to γ. Recall that the leftmost value of this graph refers to the ER set, i.e. the set of essential reactions with no growth constraints.

Notice that no reaction is mandatory to produce a null growth, hence at γ = 0, $\left|{\text{ER}}_{0}\right|$ is always 0. Furthermore, for positive values of γ the size of ${\text{ER}}_{\gamma }$ increases as the size of ${\text{RR}}_{\gamma }$ decreases. This is due to the fact that, as γ increases, some reversible reactions become non-reversible, and the flux of these reactions is necessary, i.e. essential for growth. On the other hand, as expected, the amount of growth-dependent essential reactions increases with γ.

Finally, Figure 3f shows the size of the set $R-{\text{ER}}_{\gamma }-{\text{DR}}_{\gamma }$ with respect to γ, with the leftmost point corresponding to R−ER−DR. This set is of interest as it is composed of those reactions that can contribute to growth, that is, they are not dead reactions, and at the same time are not growth-dependent essential reactions, that is, if one of these reactions is knocked out the growth can still be kept the same (notice that this does not mean that all the reactions in this set can be knocked simultaneously without affecting growth). The fact that knocking out one of these reactions does not reduce the growth might then imply the presence of redundancy in the metabolism. Therefore, this set of reactions provides the metabolism with resilience and flexibility. As it can be seen, the size of this set decreases with γ. This fact means that producing higher growths is more demanding as it requires a higher amount of reactions to sustain it. In other words, the higher the growth the more reactions are necessary to produce such growth, and therefore, less flexibility is given to the metabolism.

## 3 Implementation

CONTRABASS is a software tool for the computation of vulnerabilities in GEMs. CONTRABASS is distributed as a Python command line tool but can also be executed through an online web server at http://contrabass.unizar.es. The CONTRABASS web server is designed to offer an intuitive interface to access the operations of the tool.

The tool takes as an input a model in systems biology markup language (SBML) (Hucka *et al.*, 2019) of Levels 1, 2 or 3, and computes the set of chokepoints reactions, essential reactions, dead reactions and dead-end metabolites on the model by taking into account the dynamic constraints for the model as explained in the article. The results are then exported as a spreadsheet file and as an interactive HTML report. The operations that CONTRABASS allows include the computation of sets of chokepoints, dead, reversible, non-reversible and essential reactions with different values of γ; computation and removal of dead-end metabolites from a model; and the update of the flux bounds of the reactions according to FVA.

In addition to the above, through the interactive HTML report users can also access the data available in the model, this is, reactions, genes and metabolites along with their databases identifiers if available; explore the reaction sets defined in this article; and also explore the intersection of different sets of critical reactions.

The documentation of the tool is available at https://contrabass.readthedocs.io. CONTRABASS makes use of the Python toolbox COBRApy (Ebrahim *et al.*, 2013). The source code of the command line tool and the web server is available at https://github.com/openCONTRABASS/CONTRABASS and https://github.com/openCONTRABASS, respectively. All the code is released under GPL-3.0 License.

## 4 Discussion

The development of new drugs is an expensive and costly process. This process needs to be streamlined in order to face current health threats as those posed by MDR. In this work, computational methods to identify critical reactions, i.e. vulnerabilities, in the metabolism of microorganisms are proposed. When applied on models of pathogenic bacteria, such critical reactions are potential drug targets.

The developed methods classify the reactions of the models in different sets, e.g. essential reactions, chokepoint reactions, blocked reactions, etc., according to their role in the metabolism. In order to exploit the information available in the model as much as possible, flux- and growth-dependent sets of reactions have been defined. Such sets capture the role of the reactions as a function of the growth rate of the organism. Thus, it is possible to unveil how the cell uses its reactions to achieve its growth. The relationship among these sets has been explored and it has been shown that the set of dead reactions keeps constant for any non-optimal growth rate. Moreover, the computation of other flux- and growth-dependent sets, such as dead-end metabolites and dead reactions, can help in the identification of potential shortcomings of the model. In order to facilitate their use, an online web server and a command line tool implementing the discussed methods have been made publicly available.

## References

[btad053-B1] Abdel-Haleem A.M. et al (2018) Functional interrogation of *Plasmodium* genus metabolism identifies species-and stage-specific differences in nutrient essentiality and drug targeting. PLoS Comput. Biol., 14, e1005895. 29300748 10.1371/journal.pcbi.1005895 PMC5771636

[btad053-B2] Bannerman B.P. et al (2021) Integrated human/SARS-COV-2 metabolic models present novel treatment strategies against COVID-19. Life Sci. Alliance, 4, e202000954. 34353886 10.26508/lsa.202000954 PMC8343166

[btad053-B3] Chukualim B. et al (2008) Trypanocyc – a metabolic pathway database for *Trypanosoma brucei*. BMC Bioinformatics, 9 (Suppl 10), P5.

[btad053-B4] Ebrahim A. et al (2013) COBRApy: COnstraints-Based Reconstruction and Analysis for python. BMC Syst. Biol., 7, 1–6. 23927696 10.1186/1752-0509-7-74 PMC3751080

[btad053-B5] Folger O. et al (2011) Predicting selective drug targets in cancer through metabolic networks. Mol. Syst. Biol., 7, 501. 21694718 10.1038/msb.2011.35 PMC3159974

[btad053-B6] Gu C. et al (2019) Current status and applications of genome-scale metabolic models. Genome Biol., 20, 1–18. 31196170 10.1186/s13059-019-1730-3 PMC6567666

[btad053-B7] Heiner M. et al (2008) Petri nets for systems and synthetic biology, Vol. 5016. pp. 215–264. 10.1007/978-3-540-68894-5_7.

[btad053-B8] Henry C.S. et al (2006) Genome-scale thermodynamic analysis of *Escherichia coli* metabolism. Biophys. J., 90, 1453–1461. 16299075 10.1529/biophysj.105.071720 PMC1367295

[btad053-B9] Hucka M. et al (2019) The systems biology markup language (SBML): language specification for level 3 version 2 core release 2. J. Integr. Bioinformatics, 16, 20190021. 10.1515/jib-2019-0021 PMC6798823 31219795

[btad053-B10] Jubeh B. et al (2020) Antibacterial prodrugs to overcome bacterial resistance. Molecules, 25, 1543. 32231026 10.3390/molecules25071543 PMC7180472

[btad053-B11] Kumar M. et al (2019) Modelling approaches for studying the microbiome. Nat. Microbiol., 4, 1253–1267. 31337891 10.1038/s41564-019-0491-9

[btad053-B12] Leung E. et al; World Health Organization World Health Day Antimicrobial Resistance Technical Working Group. (2011) The WHO policy package to combat antimicrobial resistance. Bull. World Health Organ., 89, 390–392. 21556308 10.2471/BLT.11.088435 PMC3089396

[btad053-B13] Mackie A. et al (2013) Dead end metabolites-defining the known unknowns of the *E. coli* metabolic network. PLoS One, 8, e75210. 24086468 10.1371/journal.pone.0075210 PMC3781023

[btad053-B14] Mahadevan R. , Schilling C.H. (2003) The effects of alternate optimal solutions in constraint-based genome-scale metabolic models. Metab. Eng., 5, 264–276. 14642354 10.1016/j.ymben.2003.09.002

[btad053-B15] McAnulty M.J. et al (2012) Genome-scale modeling using flux ratio constraints to enable metabolic engineering of clostridial metabolism in silico. BMC Syst. Biol., 6, 1–15. 22583864 10.1186/1752-0509-6-42 PMC3495714

[btad053-B16] Murata T. (1989) Petri nets: properties, analysis and applications. Proc. IEEE, 77, 541–580.

[btad053-B17] Navid A. , Almaas E. (2012) Genome-level transcription data of *Yersinia pestis* analyzed with a new metabolic constraint-based approach. BMC Syst. Biol., 6, 150. 23216785 10.1186/1752-0509-6-150 PMC3572438

[btad053-B18] Oarga A. et al (2020) Growth dependent computation of chokepoints in metabolic networks. In: *International Conference on Computational Methods in Systems Biology, Konstanz, Germany*, pp. 102–119. Springer, Heidelberg, Germany.

[btad053-B19] Orth J.D. et al (2010) What is flux balance analysis? Nat. Biotechnol., 28, 245–248. 20212490 10.1038/nbt.1614 PMC3108565

[btad053-B20] Orth J.D. et al (2011) A comprehensive genome-scale reconstruction of *Escherichia coli* metabolism – 2011. Mol. Syst. Biol., 7, 535. 21988831 10.1038/msb.2011.65 PMC3261703

[btad053-B21] Oyelade J. et al (2018) In silico knockout screening of *Plasmodium falciparum* reactions and prediction of novel essential reactions by analysing the metabolic network. BioMed Res. Int., 2018, 8985718. 29789805 10.1155/2018/8985718 PMC5896307

[btad053-B22] Ramos P.I.P. et al (2018) An integrative, multi-omics approach towards the prioritization of *Klebsiella pneumoniae* drug targets. Sci. Rep., 8, 1–19. 30018343 10.1038/s41598-018-28916-7 PMC6050338

[btad053-B23] Richelle A. et al (2020) Towards a widespread adoption of metabolic modeling tools in biopharmaceutical industry: a process systems biology engineering perspective. NPJ Syst. Biol. Appl., 6, 6. 32170148 10.1038/s41540-020-0127-y PMC7070029

[btad053-B24] Roberts S.B. et al (2010) Genome-scale metabolic analysis of *Clostridium thermocellum* for bioethanol production. BMC Syst. Biol., 4, 1–17. 20307315 10.1186/1752-0509-4-31 PMC2852388

[btad053-B25] Thiele I. , Palsson B.Ø. (2010) A protocol for generating a high-quality genome-scale metabolic reconstruction. Nat. Protoc., 5, 93–121. 20057383 10.1038/nprot.2009.203 PMC3125167

[btad053-B26] Varma A. , Palsson B.Ø. (1994) Metabolic flux balancing: basic concepts, scientific and practical use. Nat. Biotechnol., 12, 994–998.

[btad053-B27] Vlassis N. et al (2014) Fast reconstruction of compact context-specific metabolic network models. PLoS Comput. Biol., 10, e1003424. 24453953 10.1371/journal.pcbi.1003424 PMC3894152

[btad053-B28] Yeh I. et al (2004) Computational analysis of *Plasmodium falciparum* metabolism: organizing genomic information to facilitate drug discovery. Genome Res., 14, 917–924. 15078855 10.1101/gr.2050304 PMC479120

[btad053-B29] Zomorrodi A.R. , Maranas C.D. (2012) OptCom: a multi-level optimization framework for the metabolic modeling and analysis of microbial communities. PLoS Comput. Biol., 8, e1002363. 22319433 10.1371/journal.pcbi.1002363 PMC3271020

